# Video Versus Direct Laryngoscopy for Intubation: Updated Systematic Review and Meta-Analysis

**DOI:** 10.7759/cureus.51720

**Published:** 2024-01-05

**Authors:** Saad Azam, Zainab Z Khan, Haania Shahbaz, Aisha Siddiqui, Natasha Masood, Anum, Yumna Arif, Zeenat U Memon, Muhammad Hasnain Khawar, Farina F Siddiqui, Fiza Azam, Aman Goyal

**Affiliations:** 1 Medical School, Shaikh Khalifa Bin Zayed Al-Nahyan Medical and Dental College, Lahore, PAK; 2 Internal Medicine, CMH Lahore Medical College and Institute of Dentistry, Rawalpindi, PAK; 3 Psychiatry, Dow University of Health Sciences, Karachi, PAK; 4 Medical School, Liaquat University of Medical and Health Sciences, Jamshoro, PAK; 5 Medicine, Fatima Jinnah Medical University, Lahore, PAK; 6 Medical School, Faisalabad Medical University, Faisalabad, PAK; 7 Medical School, Dow University of Health Sciences, Karachi, PAK; 8 Internal Medicine, Mayo Hospital, Lahore, PAK; 9 Orthopaedics and Trauma, Dorset County Hospital, Dorchester, Dorchester, GBR; 10 Internal Medicine, Seth Gordhandas Sunderdas Medical College and King Edward Memorial Hospital, Mumbai, IND

**Keywords:** video laryngoscopy, first pass, endotracheal intubation, direct laryngoscopy, critical care medicine, anesthesiology

## Abstract

Direct laryngoscopy (DL) is a modality commonly used in endotracheal intubation (EI). Video laryngoscopy (VL) was introduced to further facilitate the procedure with enhancement in glottic views, which captures the video image of the vocal cords to be projected onto a screen, providing enhanced visualization. This real-time video projection aids in accurately placing the endotracheal tube (ETT) through the vocal cords. In emergency and critical care settings, both laryngoscopes are used for intubations. This study assesses the efficacy of both modalities by comparing success rates in first-attempt tracheal intubation in critically ill patients.

PubMed, EMBASE, and Scopus were searched and all randomized controlled trials (RCTs) and observational studies until 2023 were included. Studies included patients in critical care settings undergoing EI under the guidance of either DL or VL. The primary outcome was the first attempt at successful tracheal intubation. The secondary outcomes assessed the comparative safety of DL and VL by comparing the rates of severe hypoxemia, severe hypotension, and cardiac arrest occurring during each modality. P-values were considered of statistical significance if below 0.05. Statistical analysis was performed using RevMan v5.4 (The Nordic Cochrane Centre, The Cochrane Collaboration, Copenhagen, Denmark). The results were displayed in the form of forest plots.

A total of eight studies were included with a total of 5348 patients, with 1780 in the DL group and 3568 in the VL group. Analysis revealed that in emergency situations, the success rate of intubation on the first attempt was significantly higher for VL than DL [81.5% vs 68%; RR= 1.19; 95% CI: 1.10, 1.29; p <0.00001; I2=70%]. There was no significant correlation between VL and severe hypoxemia [13.4% vs 11.6%; RR= 0.99; 95% CI: 0.74, 1.33; p =0.97; I2=46%], severe hypotension [6.09% vs 4.78%; RR:1.19; 95% CI: 0.83, 1.72; p =0.35, I2-15%], and cardiac arrest, [0.8% vs 0.4%; RR= 1.17; 95% CI: 0.37, 3.70]; p =0.79; I2=0%].

Our meta-analysis confirmed that VL has a higher success rate for first-pass intubation than DL. Furthermore, our analysis has shown no significant evidence linking VL to any adverse events.

## Introduction and background

Endotracheal intubation (EI), a procedure commonly performed in the intensive care unit (ICU), is crucial for patients experiencing respiratory failure and shock. It is a vital intervention to secure the airway in critical care settings [1]. However, it is noteworthy that approximately 20-30% of tracheal intubations conducted in the emergency department or ICU may encounter initial unsuccessful insertion of the endotracheal tube into the trachea. This initial failure has been associated with an increased risk of severe complications, including hypoxemia, gastric content aspiration, and bradycardia, that can potentially endanger the patient's life [2-4]. 

EI is a critical and lifesaving procedure involving the insertion of an endotracheal tube into the trachea to establish a secure airway for mechanical ventilation or anesthesia. It plays a vital role in emergencies to restore and support respiratory function. Traditionally, direct laryngoscopy (DL) has been the longstanding standard approach for endotracheal intubation [5]. During DL, the tongue and epiglottis of the patient are visualized using the laryngoscope blade, enabling direct visualization of the vocal cords through the mouth. The line of sight from the mouth then acts as a guide to inserting the endotracheal tube (ETT) through direct visualization. However, video laryngoscopy (VL) has introduced significant advancements in the field. VL follows a similar technique but with the added benefit of a camera on the distal portion of the laryngoscope blade. This camera captures the video image of the vocal cords, which can be projected onto a screen, providing enhanced visualization. This real-time video projection aids in accurately placing the ETT through the vocal cords [6]. 

The introduction of VL has revolutionized the procedure by improving visualization, accuracy, and ease of use. It offers clinicians a clearer view of the airway structures and enables better alignment and positioning of the ETT, ultimately enhancing the overall effectiveness of endotracheal intubation. Furthermore, the current guideline for prehospital emergency anesthesia with endotracheal intubation also recommends the utilization of VL [7]. A trial conducted in 2016 also demonstrated that VL exhibited a higher rate of successful first-attempt intubation in emergency situations or among patients admitted to the ICU as compared to the use of a DL [8-10]. 

This comprehensive meta-analysis aims to compare the clinical efficacy of VL and DL for emergency EI. By evaluating factors such as first-pass success rates and complications, we seek to provide evidence-based insights into the optimal technique for securing the airway in emergency and critical care settings. Such findings can guide clinical practice, improve patient safety, and ultimately contribute to better outcomes for individuals requiring endotracheal intubation in high-stakes situations.

## Review

Materials and methods 

We adhered to the Preferred Reporting Items for Systematic Reviews and Meta-Analysis (PRISMA) guidelines [11] for our study. Our trial was registered with the International PROSPERO Registry for Systematic Review and Meta-analysis with the registration number CRD42023473045.

This article was previously posted to the Research Square preprint server on the 8th of September, 2023.

Data Sources and Search Strategy

**Figure 1:**
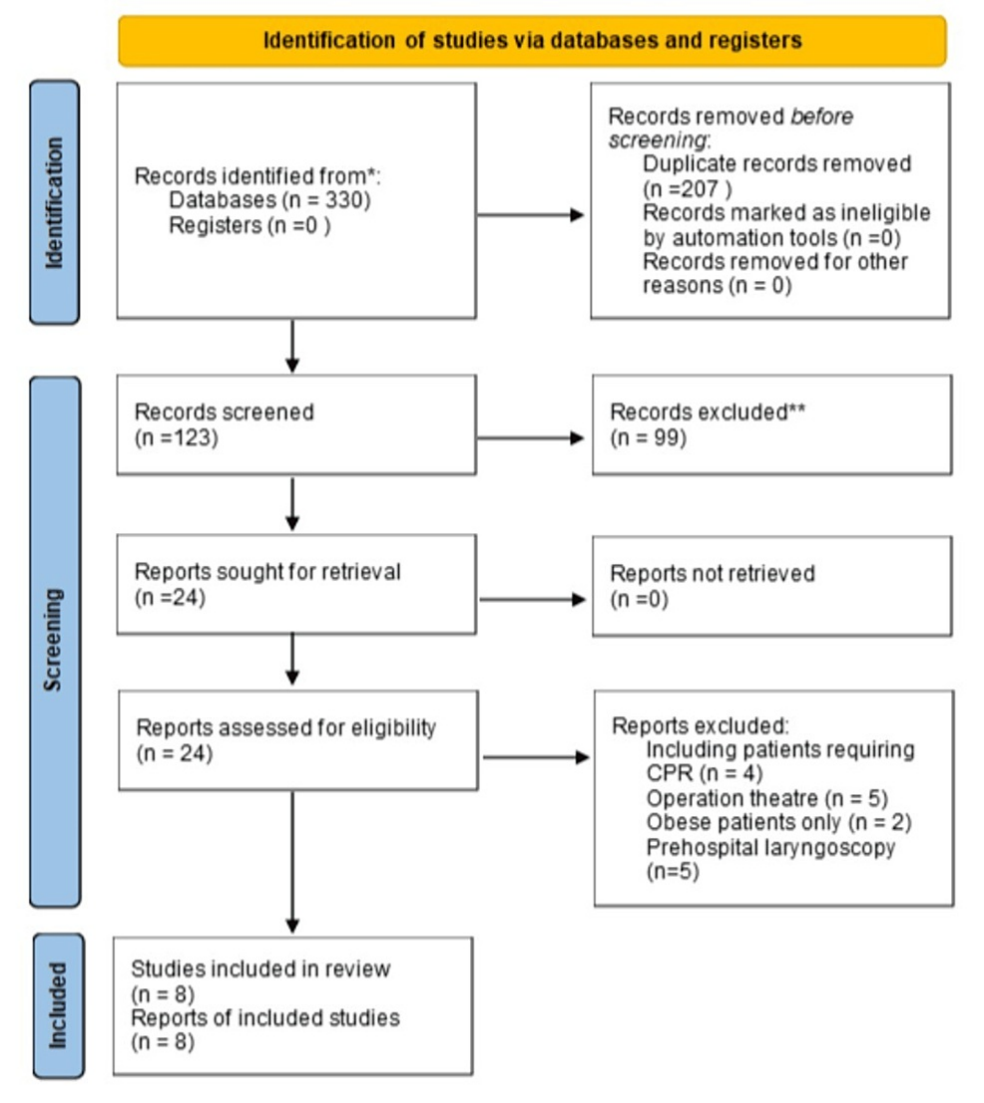
PRISMA 2020 flow diagram for new systematic reviews which included searches of databases and registers only

The PRISMA flowchart given below outlines the systematic study selection process for this meta-analysis (Figure 1). A thorough search was conducted in databases from inception to 15 October 2023. 

Two researchers (S.A. and H.S) performed a comprehensive search of various databases, including PubMed, Scopus, and EMBASE. The following keywords were used as a search string: (((Video laryngoscopy)) AND ((Direct laryngoscopy)) AND ((Tracheal intubation)) OR (Intubation)) and ((Critically ill patients)) OR ((Emergency)) OR ((Critical care))). A total of 330 records were initially identified from databases. References of retrieved trials were screened to make sure no studies were missed.

Eligibility Criteria and Outcomes

This study included prospective randomized control trials (RCTs) and observational studies that examined the differences between VL and DL in patients undergoing EI from the ICU and emergency department. The PICO (Patient/Population, Intervention, Comparison, and Outcomes) model for our research included patients in the emergency settings of critical care units, comparing VL with DL for the primary outcome of successful first-pass intubation. The study's primary outcome was the rate of successful first-attempt EI. The secondary outcomes of the study included severe hypoxemia (peripheral oxygen saturation < 80%), severe hypotension (systolic blood pressure < 65 mmHg), and cardiac arrest. The rates of occurrence of these outcomes were compared between the DL and VL groups. 

The following criteria excluded studies from the analysis: case reports, reviews, pediatric patients, and studies that used VL or DL as a rescue device in clinical settings such as ICUs and emergency departments.

Study Selection and Data Extraction

Two investigators (S.A. and M.S.) conducted independent screenings of titles and abstracts to determine eligibility. The full texts of citations that were deemed potentially relevant were thoroughly reviewed. Any disagreements during the article screening between the investigators were resolved through discussion. Two investigators independently extracted the data from the articles included. All the studies obtained from the literature search were imported to EndNote X9 (Clarivate Analytics, London, UK), and duplicates were identified and removed. This resulted in eight reports with studies that fulfilled the eligibility criteria and were included in the current version of the review. 

The following baseline data was extracted for each study: author surname, year, study design, mean patient age, intubation type, video laryngoscope type, hospital setting, and medical experts involved. A pre-piloted Microsoft Excel sheet was used for data extraction.

Quality Assessment of Included Studies

The current meta-analysis included both randomized and non-randomized studies, and the quality of the included studies was evaluated using the Cochrane risk-of-bias tool for randomized control trials, which encompasses a variety of facets, including selection bias, performance bias, attrition bias, detection bias, reporting bias, and other factors [12]. Another tool used was the Newcastle-Ottawa scale for cohort studies, which focuses on the selection criteria of studies, the comparability between groups, the exposure, and the outcomes [13]. This was accomplished by two separate researchers, whose findings were compared to eliminate inconsistencies.

Statistical Analysis

The statistical analysis was performed using Review Manager v. 5.4 (The Nordic Cochrane Centre, The Cochrane Collaboration, Copenhagen, Denmark). Risk ratios with corresponding 95% confidence intervals were calculated using a Mantel-Haenszel random effects model to present the results for each outcome. Forest plots were generated to depict the findings. Sensitivity analysis was conducted to assess the influence of individual study results on the overall conclusions. Statistical heterogeneity among the included studies was assessed using Higgins I2 statistics: a value < 25% indicates low heterogeneity; 25-75% indicates moderate heterogeneity; and > 75% indicates high heterogeneity. A significance level of p < 0.05 was used to determine statistical significance in all analyses. Funnel plots were generated for publication bias.

Results

Our analysis included eight trials; five RCTs and three observational trials. The studies included a total of 5348 patients, with 1780 in the DL group and 3568 in the VL group, and compared the success rate of the first attempt between VL and DL. 

The table below presents the characteristics of the studies [6, 10, 14-19] included in the systematic review and meta-analysis comparing VL and DL for EI in critically ill adults (Table 1). The table outlines information such as the country of study, study type, hospital setting, intubation type, VL type, medical experts involved, and age range of patients in both the VL and DL groups.

**Table 1:** Baseline Characteristics of Studies. VL= video laryngoscopy; DL= direct laryngoscopy *GlideScope, Verathon Inc., Bothell, WA ‡McGRATH™ MAC, Medtronic, Minneapolis, MN ‡Olympus America Inc., Savage, MN §Storz C-MAC (Karl Storz SE & Co. KG, Tuttlingen, Germany)

Studies	Country of Study	Study Type	Hospital Setting	Intubation Type	Video Laryngoscope Type	Medical Experts	Age, yrs (VL)	Age, yrs (DL)	Total Number
Prekker ME et al. [6]	USA	Multicenter RCT	Intensive care unit and EDs	Emergency intubations	Standard geometry blade, Hyperangulated blade	Emergency medicine resident, critical care fellow	54 (36-66)	55 (39-67)	1417
Lakticova V et al. [10]	USA	Observational	Intensive care unit	Emergency intubations	GlideScope*	Pulmonary critical care experts and intensive care unit	66 16.96	69 16.88	392
De Jong A et al. [14]	France	Observational	Intensive care unit	Emergency intubations	McGRATH MAC†	ICU physicians and anesthetists	63 (55-70)	59 (49-69)	210
Silverberg MJ et al. [15]	Israel	Randomized clinical trial	Intensive care unit	Emergency intubations	GlideScope	Pulmonary and critical care experts	65.4	69.6	117
Janz DR et al. [16]	USA	Randomized clinical trial	Intensive care unit	Emergency intubations	McGRATH/ GlideScope/ Olympus‡	Pulmonary experts and critical care supervised by physicians	59 (49-68)	60 (51-67)	150
Hypes C et al. [17]	USA	Propensity matched analysis	Intensive care unit	Emergency intubations	Multiple	6 doctors (postgraduate)	59 (49-69)	60 (53-73)	905
Lascarrou JB et al. [18]	France	Randomized clinical trial	Intensive care unit	Emergency intubations	McGRATH MAC	ICU physicians	62.7 (15.3)	62.8 (16.3)	371
Prekker ME et al. [19]	USA	Post hoc analysis of data from 2 multicenter RCT	Intensive care units and EDs	Emergency intubations	Standard geometry (Storz C-MAC§, McGRATH MAC, Glidescope Macintosh shape) and Hyperangulated (Storz D blade, Glidescope traditional shape) Macintosh-style blade was favored (84.1%) over a hyperangulated blade (15.9%).	Residents, Fellows, Attending physicians	60 (47-70)	59 (47-70)	1786

Quality Assessment and Publication Bias

The quality assessment of the studies included in this meta-analysis revealed a range of study qualities, with some demonstrating robust methodologies and low risk of bias, while others had limitations. The assessment utilized predefined criteria and tools such as the Newcastle-Ottawa Scale for observational studies (Table 2) and the Cochrane risk-of bias tool for RCTs (see Appendices). Regarding publication bias, the analysis indicated the possibility of selective reporting and publication of studies, which could introduce bias in the overall findings. These assessments emphasize the importance of considering the quality of included studies and the potential impact of publication bias on the meta-analysis results.

**Table 2:** Newcastle–Ottawa Quality Assessment Scale (NOS) for Cohort Studies Newcastle Ottawa scale for assessment of bias of cohort studies: A study can be given a maximum of one star for each numbered item within the Selection and Outcome categories. A maximum of two stars can be given for comparability. Good quality: 3 or 4 stars in selection domain AND 1 or 2 stars in comparability domain AND 2 or 3 stars in outcome/exposure domain. Fair quality: 2 stars in selection domain AND 1 or 2 stars in comparability domain AND 2 or 3 stars in outcome/exposure domain. Poor quality: 0 or 1 star in selection domain OR 0 stars in comparability domain OR 0 or 1 stars in outcome/exposure domain.

Authors (Year)	Study type	Selection				Comparability	Outcome			Final score
		Representativeness of the exposed cohort	Selection of the non-exposed cohort	Ascertainment of exposure	Demonstration that outcome of interest was not present at the start of the study	Comparability of cohorts on the basis of the design or analysis	Assessment of outcome	Was follow-up long enough for outcomes to occur	Adequacy of follow-up of cohorts	
Lakticova A. et.al (2015) [10]	Cohort	*	*	*	*	**	*			7
Hypes C et.al (2016) [17]	Cohort	*	*	*	*	**	*	*	*	9
De Jong A et.al (2013) [13]	Cohort	*	*	*	*	**	*			7

Primary Outcome

**Figure 2:**
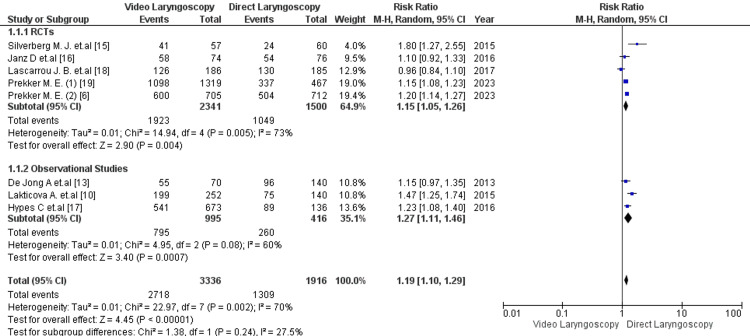
Forest plot comparing first-pass intubation success rates in critical care patients with video laryngoscopy vs direct laryngoscopy. RCT=randomized controlled trial P-value < 0.05 is considered to be of statistical significance.

All eight studies included reported the primary outcome of first-pass success rate [6, 10, 14-19]. The pooled analysis showed a significantly higher rate of first-pass intubation with VL than DL [81.5% vs 68%; RR= 1.19; 95% CI: 1.10, 1.29; p <0.00001; I2=70%] (Figure 2). The heterogeneity test indicated high heterogeneity among studies (I2 = 70%).

Secondary Outcomes

**Figure 3:**
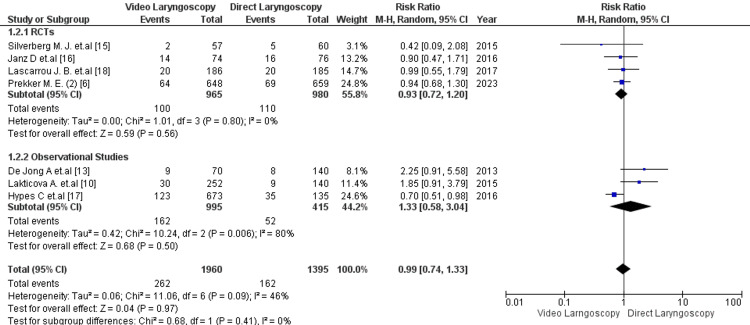
Forest plot comparing the adverse effect of severe hypoxemia in critical care patients with video laryngoscopy vs direct laryngoscopy. RCT=randomized control trial P-value < 0.05 is considered to be of statistical significance.

**Figure 4:**
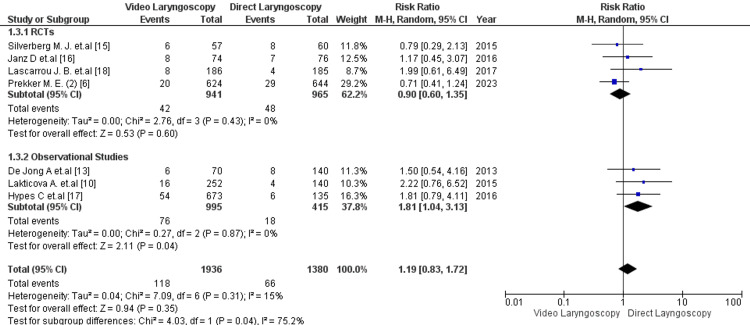
Forest plot comparing the adverse effect of severe hypotension in critical care patients with video laryngoscopy vs direct laryngoscopy. RCT=randomized control trial P-value < 0.05 is considered to be of statistical significance.

**Figure 5:**
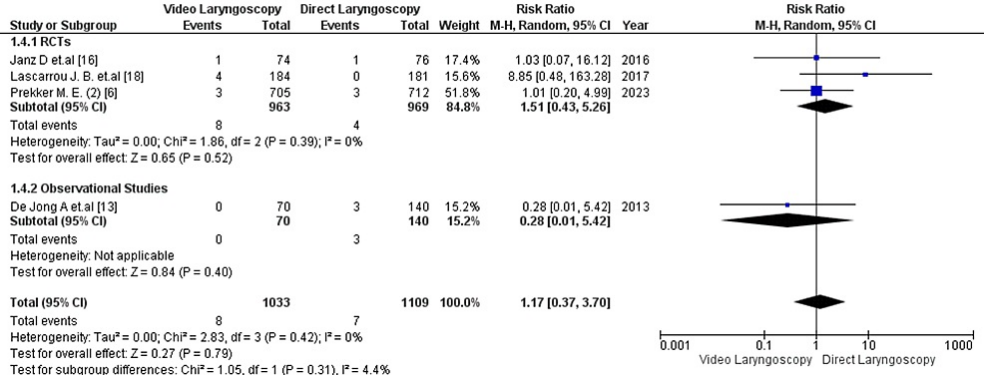
Forest plot comparing the adverse effect of cardiac arrest in critical care patients with video laryngoscopy vs direct laryngoscopy. RCT=randomized control trial P-value < 0.05 is considered to be of statistical significance.

Seven studies reported the outcome of severe hypoxemia following intubation [6, 10, 14-18]. The analysis did not show a statistically significant difference in the incidence of severe hypoxemia between VL and DL [13.4% vs 11.6%; RR= 0.99; 95% CI: 0.74, 1.33; p =0.97; I2=46%] (Figure 3). The studies showed moderate heterogeneity. Seven studies shared the outcome of severe hypotension following intubation [6, 10, 14-18] There was no significant correlation between the VL and DL groups for the risk of developing severe hypotension [6.09% vs 4.78%; RR:1.19; 95% CI: 0.83, 1.72; p =0.35, I2-15%] (Figure 4). Low heterogeneity was reported among the studies. Additionally, 4 studies reported the outcome of cardiac arrest following EI [6, 14, 16, 18]. The risk of developing cardiac arrest for VL was not significantly higher than for DL [0.8% vs 0.4%; RR= 1.17; 95% CI: 0.37, 3.70]; p =0.79; I2=0%] (Figure 5). No heterogeneity was observed among the studies.

Discussion

Our results demonstrated a significantly increased rate of first-pass intubation with VL compared with DL in patients in the ICU. Moreover, the risk of adverse events, such as severe hypotension, severe hypoxemia, and cardiac arrest, was comparable between the two groups.

Airway management in critically ill individuals in non-operative settings presents significant challenges and is frequently associated with potentially life-threatening complications. Notably, the incidence of difficult EI in the ICU surpasses that observed in the operating room [20].

EI is one of the most frequently performed procedures in the ICU. Video laryngoscopes, which are said to provide an enhanced method of viewing the glottis, as shown in the study by Prekker et al. [19], are recommended to enhance airway management within the ICU, particularly for patients presenting with warning signs of a possibly difficult intubation, which could include a Mallampati score of III where the hard and soft palate are visible, but the uvula is somewhat obscured, and score IV, where only the hard palate is visible. In addition, obstructive sleep apnea, restricted mobility of the cervical spine, difficulty in mouth opening, coma, severe hypoxemia, and inexperienced operators are all risk factors for difficult EI [21]. Importantly, the first-pass success rate is key to reducing the incidence of complications and possibly increasing mortality [22]. In 2013, De Jong et al. studied the use of a combination of VL and DL and found that the former was associated with significantly higher first-pass intubation and better visualization of the glottis [14]. A study by Lakticova et al. in 2015 [10] also found failure rates for difficult EI to be reduced when using VL compared to DL; in addition, the rate of EI was significantly reduced in the former group. However, they reported a statistically non-significant increase in hypoxemia in the VL group, an effect also reported by Lascarrou et al. [18], possibly because of the increased duration of the intubation procedure with VL. However, hypoxemia after intubation was also similar in both groups in our meta-analysis (Figure 3). Lascarrou et al. also reported a significantly higher incidence of severe, life-threatening complications with VL, whereas the time taken for insertion and the number of attempts required for successful intubation were all statistically better when VL was used rather than DL. Silverberg et al. found increasing attempts to intubate to be associated with higher complication rates [15], a finding also noted by Hypes et al. [17], who reported significantly increased odds of a complication, which included desaturation, most noticeably, but also aspiration, airway trauma, laryngospasm, bradycardia, and cardiac arrest, with the last one being analyzed in our own study, with results concluding no differences in such incidences in the two groups. Two studies supported these findings; Janz et al. compared VL and DL for EI in critically ill adults and failed to demonstrate an increase in intubation on the first attempt with VL [16], a fact that was attributed to previous studies possibly being confounded by bias, as well as the infrequent use of neuromuscular blockade during DL as per the protocol, an intervention that actually resulted in a greatly increased success rate via DL. The second study by Prekker et al. [6] compared VL and DL for EI in critically ill adults and similarly demonstrated no significant differences in the occurrence of complications. The study did, however, demonstrate an increase in intubation on the first attempt with VL. 

Hence our study holds value as it presents a novel analysis of the success of first-pass intubation when done with VL as compared to DL, with comparable risks between the two.

Limitations

This meta-analysis has several limitations that should be considered when interpreting the findings. First, the included studies exhibited heterogeneity that may have influenced the overall results. Variations in the study settings, types of laryngoscopes used, and patient characteristics could have contributed to this heterogeneity, potentially affecting the generalizability of the findings. Additionally, the brand of the video laryngoscope used, the bulkiness of the equipment, and the blade shape are all variables that may alter the findings above. It is crucial to highlight that the likelihood of bias in the incorporated studies could significantly affect the precision of the results. Second, the assessment of the study quality revealed varying degrees of methodological limitations and potential sources of bias, which may have influenced the overall outcomes. Third, the assessment of publication bias indicated the potential for selective reporting and publication of studies, thereby introducing the possibility of bias in the overall conclusions. Unpublished studies or those with negative results may have been missing from the analysis, potentially affecting the overall findings. Finally, the generalizability of the findings is limited to critically ill patients and emergency orotracheal intubation settings. The results may not be directly applicable to other patient populations or in cases of non-emergency intubation. Further research is needed to address these limitations and to provide more comprehensive insights into the effectiveness and safety of VL versus DL in diverse clinical scenarios.

## Conclusions

VL provides higher success rates for first-pass intubation than DL. The risk of complications, such as severe hypoxemia, severe hypotension, and cardiac arrest, was comparable between the two groups. Our findings corroborate the superiority of VL over DL in EI, with a higher first-pass intubation success rate. Ultimately, it is important to comprehensively understand the advantages and limitations of both VL and DL to make informed decisions in clinical practice. With factors such as cost effectiveness to consider, implementation and usage of VL may vary from setting to setting. Further large-scale RCTs are needed to generate real-world evidence to demonstrate further advantages of VL over DL before large scale implementation can be discussed.

## Figures and Tables

**Figure 6:**
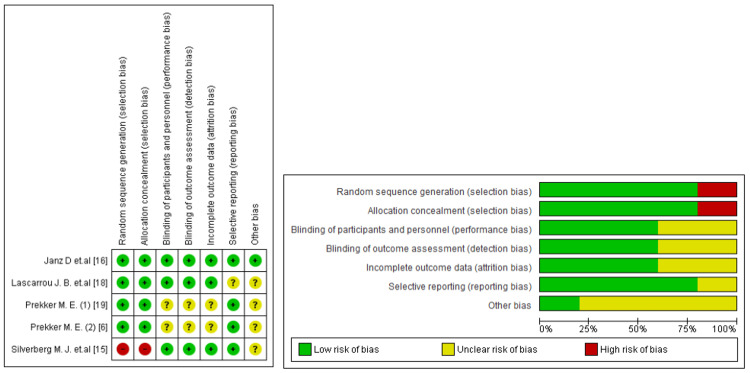
Results obtained using Cochrane's risk-of-bias tool (v.2.0) for reporting risk assessments of randomized controlled trials '+' sign= Positive indication of the application of a particular method for reduction of bias as part of the research protocol. '-' sign= Negative indication of the application of a particular method for reduction of bias as part of the research protocol. '?' sign= Unclear/uncertain indication of the application of a particular method for reduction of bias as part of the research protocol.

## Appendices

Figure 6 shows the results of the analysis performed using the Cochrane risk-of-bias tool for randomized trials.
